# Geographic patterns of seed trait variation in an invasive species: how much can close populations differ?

**DOI:** 10.1007/s00442-021-04971-2

**Published:** 2021-07-03

**Authors:** Erola Fenollosa, Laia Jené, Sergi Munné-Bosch

**Affiliations:** 1 Department of Evolutionary Biology, Ecology and Environmental Sciences, Faculty of Biology, Universitat de Barcelona, Avinguda Diagonal 643, 08028 Barcelona, Spain; 2 Institute of Research in Biodiversity (IRBio-UB), Universitat de Barcelona, Avinguda Diagonal 643, 08028 Barcelona, Spain

**Keywords:** Bioclimatic variables, *Carpobrotus edulis*, Geographic distance, Longevity, Mediterranean

## Abstract

Seeds play a major role in plant species persistence and expansion, and therefore they are essential when modeling species dynamics. However, homogeneity in seed traits is generally assumed, underestimating intraspecific trait variability across the geographic space, which might bias species success models. The aim of this study was to evaluate the existence and consequences of interpopulation variability in seed traits of the invasive species *Carpobrotus edulis* at different geographical scales. We measured seed production, morphology, vigour and longevity of nine populations of *C. edulis* along the Catalan coast (NE Spain) from three differentiated zones with a human presence gradient. Geographic distances between populations were contrasted against individual and multivariate trait distances to explore trait variation along the territory, evaluating the role of bioclimatic variables and human density of the different zones. The analysis revealed high interpopulation variability that was not explained by geographic distance, as regardless of the little distance between some populations (< 0.5 km), significant differences were found in several seed traits. Seed production, germination, and persistence traits showed the strongest spatial variability up to 6000% of percent trait variability between populations, leading to differentiated *C. edulis* soil seed bank dynamics at small distances, which may demand differentiated strategies for a cost-effective species management. Seed trait variability was influenced by human density but also bioclimatic conditions, suggesting a potential impact of increased anthropogenic pressure and climate shifts. Geographic interpopulation trait variation should be included in ecological models and will be important for assessing species responses to environmental heterogeneity and change.

**Supplementary Information:**

The online version contains supplementary material available at 10.1007/s00442-021-04971-2.

## Introduction

Seeds, the reproductive units of flowering plants, constitute one of the main factors defining the persistence and expansion of species and therefore play a crucial role in determining species success (Saatkamp et al. 2014; Gioria and Pyšek 2016). Moreover, seed persistence and dispersal have deep consequences for a species genetic diversity and adaptative dynamics (Tigano and Friesen 2016). A wide range of morphological and physiological traits enable seeds to coordinate persistence and germination timing, as well as dispersion maximization through different agents (Poschlod et al. 2013; Long et al. 2015; Saatkamp et al. 2019). Variations in seed morphological traits may determine differential dispersion strategies that can favour adaptation and colonization of different habitats (Lamberti-Raverot et al. 2019). For instance, high seed production, small seed mass, and rapid germination have been associated with higher invasive success in novel habitats (Pyšek and Richardson 2007; Gioria and Pyšek 2017; Gioria et al. 2018). In species with orthodox seeds, viable seeds may enter dormancy, a temporal suppression of germination under favourable conditions, thanks to their ability to tolerate considerable desiccation (Bewley et al. 2013). Higher dormancy rates may facilitate the formation of a persistent seed bank, which may substantially contribute to determining the invasion potential, given the role of seed banks as sources of propagules, genetic diversity, and in spreading the risk of germination failure over time (Gioria et al. 2019). Complex biochemical regulation prevents seeds to germinate, resisting for several years at the soil seed bank (Kucera et al. 2005). Seed longevity may differ between species, ranging from just a few months to more than 2000 years in *Phoenix dactylifera* (Sallon et al. 2008). Even within a species, seed longevity may vary based on several factors such as moisture content, relative humidity, oxygen pressure, and temperature (Walters et al. 2005). Longevous seeds may increase the capacity of a species to persist in a particular site and increase the impact of invasive species due to increased needs of post-eradication management (Gioria et al. 2016). Altogether, seed morphology, production, viability, germination, biochemistry, and persistence may have important implications in species success (Saatkamp et al. 2019), as they offer an approximated reflect of the soil seed bank dynamics (Gioria and Pyšek 2016).

Despite all well-known seed processes and the role of seeds on species persistence and expansion, the degree of variability that a species may show in seed production, viability, germination and persistence between different closer and distant populations may be underestimated. Intraspecific variation of seed traits still constitutes a major concern in the research agenda for seed-trait functional ecology (Saatkamp et al. 2019) as the reliability of trait-based mechanistic models depends on the representation of intraspecific trait variation, that may drive to enormous differences (Albert et al. 2011). The understanding of the causes, consequences, and degree of intraspecific variability may impact ecological models of species response to global change and species potential distribution (Saatkamp et al. 2019; Snell et al. 2019).This may be particularly important for understanding and modelling the biological invasion process, where modelling seed persistence and expansion across the geographic space (i.e., across different populations) is crucial for risk assessment and management. In this way, Jarić et al. (2019) highlighted that spatial variation in seed traits may be one of the contributors to the uncertainty when trying to model invasive species impact and develop management strategies. Not only for improving ecological modelling, but variability on an invasive species impact may require the development of differential management strategies (Januchowski-Hartley et al. 2018).

To what extend a species seed traits dissimilarity increases with geographic distance is crucial in assumptions taken for species persistence and expansion modelling. However, the geographic variation of seed traits for a species, i.e., seed traits interpopulation variability, may come from different sources. Environmental heterogeneity, which includes climatic and habitat variability, is considered the main driver of intraspecific variability (Kuppler et al. 2020) and may strongly contribute to interpopulation variability with the more similar populations under the more similar environmental conditions. It may be therefore assumed that geographic distance together with environmental distance (environmental dissimilarity) and habitat heterogeneity may determine interpopulation variability. Bioclimatic dissimilarity was key in determining seed thickness variation of the invasive plant *Taraxacum officinale* in five differentiated Chilean populations with a rainfall gradient (Molina-Montenegro et al. 2018), but for example, the study from Bogdziewicz et al. (2019) found that *Quercus petraea* seed production may vary across populations independently of the geographic (and bioclimatic) distance between them. The bioclimatic conditions that promote increased interpopulation variability are not clear and may not be universal. It has been proposed that abiotic conditions near the limit of tolerance of a species can exert a role towards both increased or reduced intraspecific variability (Caruso et al. 2017; Helsen et al. 2017). Not only the climatic conditions a species may encounter in a location but also habitat heterogeneity may contribute to variability in the environment and modulate species intraspecific variability (De Smedt et al. 2018). Urbanization is a land-use change form characterized by increased human impacts as a consequence of their increased presence, fragmentation of natural habitats, intensive land transformation and an increase in impervious surface land cover thus reducing the spatial extent for vegetation (Irwin and Bockstael 2007; Williams et al. 2015). Urban environments create a wide range of novel habitats such as industrial areas or waste dumps that may constrain species performance and community assemblance (Irwin and Bockstael 2007), and therefore increased intraspecific variability in urban species traits may be expected. This may be particularly important in invasive species, that benefit from those novel habitats within urban areas and are subjected to novel environmental pressures that may induce high intraspecific variation, especially regarding plant seed traits that compromise species persistence and expansion (Gaertner et al. 2017). Moreover, multiple introductions and therefore the increased propagule pressure in urbanized areas with higher human presence may also propitiate higher intraspecific variability in invasive species (Smith et al. 2020).

The invasive species *Carpobrotus edulis* (L.) N. E. Br. (Aizoaceae) is a mat-forming trailing succulent perennial native from South Africa that has been introduced in all continents, strongly impacting Mediterranean regions (Vilà et al. 2008). *C. edulis* was introduced in Europe as a valuable ornamental plant but also for soil and dune stabilization (Preston and Sell 1988). *C. edulis* impacts reside on its ecosystem engineer capacity to modify the surrounding environment by altering physicochemical soil properties (Molinari et al. 2007; Novoa et al. 2013) and decrease native plant richness and functional diversity (Jucker et al. 2013; Novoa and González 2014). *C. edulis* has small hard-coated reniform orthodox seeds, which can generate a permanent soil seed bank that may contribute to the species impact and persistence in the invaded communities (Chenot et al. 2014; Fenollosa et al. 2020). *Carpobrotus sp.* seeds may persist in the soil seed bank for more than 5 years allowing quick reinvasion many years after removal (up to 8 years) (Affre 2011; Ruffino et al. 2015). In Catalonia, *C. edulis* constitutes a major biodiversity conservation concern because it inhabits and impacts most of the coastal zones. However, the shoreline has been massively urbanized, leaving few spaces for vegetation. Across the Catalan coast, some zones have been urbanized more than others. In the northern part, the zone of Cap de Creus (CA) is the eastern foothill of the Pyrenees. It includes marine and terrestrial protected areas and constitutes an area of high biological, geological, and landscape quality with large extensions of woody sclerophyll Mediterranean vegetation. Forty kilometres south, the zone of middle Costa Brava (CB, the Baix Empordà county) constitutes an area where tourism is more intense and combines large zones of established plant communities coexisting with urbanizations, campings and touristic complexes. Finally, getting closer to Barcelona, the Maresme (M) coast is intensely urbanized with large agricultural areas leaving almost no virgin plant communities. Among those three zones, there is a strong gradient in human presence in terms of population density per squared kilometre. High phenotypic plasticity has been observed in this invader regarding biomass allocation, morphological variations and photoprotective responses (Traveset et al. 2008; Roiloa et al. 2014, 2016; Fenollosa et al. 2017). However, seed traits variability across different geographic scales in the differentiated three zones that *C. edulis* inhabits in Catalonia has not been studied and may determine differential invasive vigour in this highly plastic invasive species.

The aims of this study were to (a) test the existence of interpopulation variability in plant traits associated with sexual reproduction of the invasive species *C. edulis* at different geographical scales, contrasting closer (< 4 km) and distant (> 40 km) populations, and (b) determine the role of geographic distance, bioclimatic conditions and human presence on interpopulation variability. We hypothesized that (1) the variability found within a population (intrapopulation) may be lower than the variability between different populations (interpopulation) in distant populations (> 40 km), but not in closer populations (< 4 km), so geographic distance may have a role determining similarity between populations due to similar environmental conditions. Moreover, we hypothesized that (2) distant populations may differ in key seed traits that may determine differentiated population dynamics such as: seed production, viability, germination and dormancy, as a consequence of local adaptation. This would hamper invasive species dynamics modelling, as a model obtained from the mean values of different populations would not reflect the actual species dynamics. Finally, we expected (3) increased seed traits variability in zones with increased human presence due to the increased propagule pressure and the variability in microhabitat conditions, and higher seed traits variability in sites where individuals of this species might suffer more stressful conditions according to previous studies (lower temperatures on winter and lower water availability on summer) (Fenollosa et al. 2017). To test these hypothesis, we evaluated the interpopulation variability of nine populations of *C. edulis* and measured different parameters describing different aspects of the invasive species reproductive traits such as: seed morphology, production, viability, germination, biochemistry and persistence analysed the variability within and between the different populations at contrasted geographic distances in relation with their degree of human presence and bioclimatic variables.

## Materials and methods

### Plant populations and seed collection

**Fig. 1:**
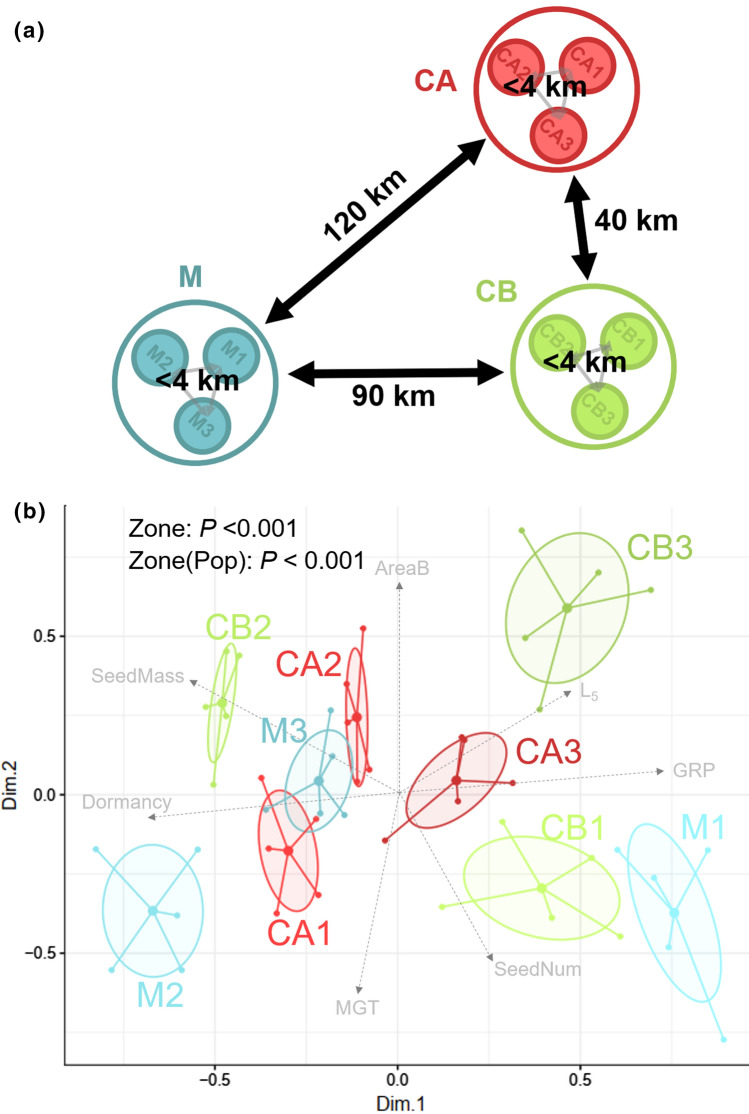
**a** Relative location of the nine studied populations (filled circles) of *C. edulis* distributed in three differentiated zones: Maresme (M), Costa Brava (CB) and Cap de Creus (CA). **b** Results of multidimensional scaling analysis (MDS) evaluating differences in nine seed traits among studied populations. Traits indicated in grey have significant (*P* < 0.01) contribution population variability. Ellipses represent 95% of confidence intervals. *P*-values correspond to PERMANOVA results for Zone and Population (nested in Zone) factors

Nine populations of *C. edulis* along the Catalan coast (NE Spain) were selected considering different geographic distances (Fig. 1a). Within three distant zones (> 40 km) (Cap de Creus, Costa Brava and Maresme) where *C. edulis* is abundant, three populations were sampled based on closeness (< 4 km) and enough fruit production to measure all seed traits. In average, the three *C. edulis* populations in the CA zone have a population density of 39 habitants km^−2^, whereas CB has 188 habitants km^−2^ and M 285 habitants km^−2^ (Geostat Eurostat 2011). These differences in human presence and habitat availability (higher in the CA zone) may also influence the surrounding vegetation, besides terrain characteristics. Unfortunately, there is no data regarding introduction time or invasion front within the territory.

From each population of a minimal area of 500 m^2^, within the period of natural seed rain, 600 fruits were collected to ensure representativeness. Fruits were opened and all the obtained seeds were pooled (more than 10,000 seeds per population). Seeds were kept at darkness and room temperature until analysis (3–4 weeks). Multiple ecological significant traits related to seed production, seed morphology, viability, germination, persistence, and biochemistry were measured and are summarized in Online Resource 2.

### Bioclimatic data

Nineteen bioclimatic variables were obtained from the WorldClim 2 database at 0.5 arcmin resolution (∼1 km^2^) (Fick and Hijmans 2017). The bioclimatic variables represent annual trends (e.g., mean annual temperature, annual precipitation) seasonality (e.g., annual range in temperature and precipitation) and extreme or limiting environmental factors (e.g., temperature of the coldest and warmest month, and precipitation of the wet and dry quarters). Climatic information of all studied populations can be found in the Online Resource 1.

### Seed morphology and production

Five fruits from each population were used to determine seed production in terms of fruit weight (FruitWeight), total seed weight (SeedsWeight) and total seed number (SeedNum). From each fruit, five seeds were randomly selected to measure morphometric seed parameters such as seed area, perimeter, major diagonal, minor diagonal, and thickness, considering basal and lateral scaled captures of 4 × magnification under a binocular loupe leading to the following parameters from basal capture: basal seed area (AreaB), basal seed perimeter (PerimB), major basal diagonal (MajorB), minor basal diagonal (MinorB), and from lateral capture: lateral seed area (AreaL), lateral seed perimeter (PerimL), major lateral diagonal (MajorL), minor lateral diagonal (MinorL). All measurements were performed with ImageJ software (Wayne Rasband, Java, National Institutes of Health, Bethesda, USA). The five seeds per fruit were averaged and considered individual replicates, with one value per fruit for each seed morphology trait, to contrast these values with seed production traits per fruit unit.

Five replicates of fifty seeds were used to estimate seed mass (SeedMass) and the dry seed mass (DrySeedMass) after four days at 70 °C. Seed mass and dry seed mass were obtained by dividing the obtained fresh and dry weight respectively by seed number.

### Seed vigour: germination, viability and longevity

Seed germination for all populations was performed as described in Fenollosa et al. (2020) with 12 h photoperiod and temperature alternance between 25 °C during day and 10 °C at night. Germination was assessed every three days on petri plaques with fifty seeds each with five replications per population. Total germination percentage (GRP) was estimated after curve saturation at 70 days. Different seed germination indexes regarding speed and synchrony were estimated: mean germination time (MGT) (days), mean germination rate (MGR) (seeds day^−1^), germination speed percentage (GSP) (%), uncertainty index (UNC) (bits), synchronization index (SYN) (from 0 to 1), germination variance (VGT), germination standard deviation (SDG), coefficient of variation (CVG) (%), and T_50_ (The time required for 50% germination). The germination uncertainty index (UNC) is based on the Shannon indexes and estimates the uncertainty in predicting the informal entropy associated with the distribution of the germination relative frequency in bits units (information units) (Labouriau and Valadares 1983). The synchronization index (SYN) assesses the synchrony of one seed with other included in the same replication (SYN = 0 when at least two seeds germinate one each time and SYN = 1 when the germination of all the seeds occurs at the same time) (Ranal and Santana 2006). Five replications per population, including fifty seeds per replication were used for the germination test.

Seed viability was assessed using the viability tetrazolium (Triphenyl tetrazolium chloride) test following the method and embryo viability classification described in Fenollosa et al. (2020) for *C. edulis*. In brief, after 24 h of seed imbibition, seeds were delicately pierced to ensure tetrazolium penetration. Seeds with 0.1% tetrazolium (Sigma-Aldrich, Steinheim, Germany) were incubated for 48 h at 40 °C before viability assessment. Six different categories were used to classify embryo status: viable (totally stained), weakly (pale stained), patchy (partially stained), patchy-weakly (partially pale stained), dead (white embryo), and aborted (no embryo found). The sum of weakly, patchy and patchy-weakly seeds constitutes low vigour seeds without survivance probabilities (Dying). The percentage of dormant seeds was estimated as the difference between viable but not germinated seeds.

Seed longevity was assessed through an accelerated ageing test as described by Fenollosa et al. (2020) for *C. edulis* on which seeds were subjected to 55 °C under high relative humidity conditions (80–90%) for different timings that lead to a progressive viability loss. Viability was totally loss for all populations after 196 h under the accelerated ageing conditions and L_5_ (resistance to deterioration), L_50_ (medium longevity) and L_95_ (lethal ageing time) (Number of hours to lose 5, 50, and 95% viability respectively) were used as longevity estimators. Five replicates per population, including 250 seeds per replication were used for viability and longevity assessment.

### Seed biochemistry: antioxidants and seed water content

During ageing, seeds are subjected to oxidative stress that may be counterbalanced with antioxidants compromising seed longevity (Bailly 2004). Among antioxidant systems, tocochromanols are lipophilic antioxidants particularly abundant in seeds that were found to be essential in determining seed longevity (Sattler et al. 2004). Tocochromanols are lipid-soluble antioxidant molecules and its accumulation in seeds has been described to be critical in maintaining seed viability by protecting lipids from oxidation during germination and early seedling growth (Sattler et al. 2004). Five replicates of 100 mg of seed samples from each population were ground in liquid nitrogen using a mix ball and extracted with cold methanol containing 0.01% butylated hydroxyltoluene using ultrasonication. After centrifuging at 14,000 g for 10 min at 4 °C, the supernatant was collected and the pellet re-extracted with the same solvent until it was colourless; then, supernatants were pooled and filtered with 0.22 μm and transferred to high-performance liquid chromatography (HPLC) vials. Tocochromanols were separated isocratically in a normal-phase HPLC system and quantified with a fluorescent detector, as described by Amaral et al. (2005). Quantification was based on the results obtained from the fluorescence signal and compared to that of a calibration curve made with authentic standards (Sigma-Aldrich, Steinheim, Germany). Gamma-tocopherol (γ-Toc), alpha-tocopherol (α-Toc) and their sum (Toc) were detected in *C. edulis* seeds.

Seed water content (WC), and imbibed seed water content (WC_Imb) were measured with five replicates of fifty seeds per population as (fresh − dry weight × 100) × dry weight^−1^ from seeds and imbibed seeds for 24 h respectively. Dry weight was obtained after four days at 70 °C.

### Data analysis

All analyses were performed in R 4.0.3 (R Core Team, 2020) using the following R packages: vegan (Oksanen et al. 2019), mctoolsr (https://github.com/leffj/mctoolsr/), multcomp (Hothorn et al. 2008), agricolae (Mendiburu and Yaseen 2020) and VCA (Schuetzenmeister and Dufey 2020). Germination parameters were calculated using *GerminaR* package (Lozano-Isla et al. 2019).The *ggplot2* R package (Wickham 2016) and SigmaPlot 10.0 (Systat, USA) were used for plotting.

#### Aim A: interpopulation vs. intrapopulation variability

To evaluate the degree of interpopulation variability (aim A), the percent potential variability of each trait between populations, hereafter ‘percent trait variability between populations’ was calculated as the mean percent difference when contrasting all measured values from one population to another.

To test the similarity of the different populations and contrast the hypothesis that intrapopulation variability is lower than interpopulation variability (hypothesis 1), nine traits were selected. Trait selection was based on their ecological relevance and significant contribution to explaining the global dataset variance in the multivariate space using multidimensional scaling. The nine selected traits represent the 6 trait types considered: basal seed area and seed mass (seed morphology), seed number per fruit (seed production), seed viability (viability), seed germination and mean germination time (germination), γ-tocopherol (biochemistry), resistance to deterioration L_5_ and dormancy (persistence). Permutational multivariate analysis of variance (PERMANOVA) was used to contrast zones and populations (nested in zone) using a multidimensional approach with 999 permutations with scaled data, using the *adonis()* function from the *vegan* R package, using the scaled trait values as a response variable and Zone and Zone(Pop) as predictor variables. The function *calc_pairwise_permanovas()* from the R package *mctoolsr* was used to evaluate differences between zones and populations. As measured traits resulted in independent replicates, PERMANOVA results were tested after multiple dataset randomizations to confirm the obtained statistic results.

To evaluate the populations' differentiation in individual seed traits and test the hypothesis that distant populations may differ in key seed traits that may determine differentiated population dynamics (hypothesis 2), a nested ANOVA was used to evaluate the significance of the factors Zone and Population (nested in Zone) in all measured seed traits using the trait values as a response variable and Zone and Zone(Pop) as predictor variables. ANOVA assumptions were tested with Shapiro–Wilk and Levene tests for normality and homoscedasticity. The Tukey test was used as a post-hoc method using the *multcomp* and *agricolae R* packages to evaluate differences between populations for all traits.

#### Aim B: the role of geographic distance, bioclimatic conditions and human density on interpopulation variability

To determine the main source of variation and determine the role of geographic distance, bioclimatic conditions and human density on interpopulation variability (aim B), relative variance decomposition at the population and zone-levels was performed for the nine selected traits using the *anovaMM()* function from the *VCA* R package. For each trait (response variable), variance was decomposed and quantified across sampling scales and expressed as a percentage of the total variance explained by Zone, Zone(Pop) and residual variance (predictor variables).

To determine the role of geographic distance, Mantel tests were run to evaluate the correlation between distance matrices of individual seed traits and geographic distance using the *mantel()* function from the *vegan* R package, using 999 permutations. This allowed testing the second part of hypothesis 1 regarding the role of geographic distance determining populations' similarity.

To assess the role of bioclimatic variables and human density in interpopulation variability within a zone (hypothesis 3), the *betadisper()* function from the *vegan* R package was used to obtain the ‘distance to centroid’, hereafter: zonal variability, for the multidimensional data of each zone, this is, the distance of each data point to the centroid obtained using the scaled data of the 9 selected traits of each zone. This parameter was used to compare trait variation within each of the three distant zones. An ANOVA was performed to test significant differences between the response variable zonal variability in the different Zones. The Tukey test was used as a post-hoc method. Zonal variability was correlated with the data of all 19 bioclimatic variables in each population and the data of human density in each Zone, using the *cor()* function in R. Due to the high number of comparisons and to avoid type I error, *P-*values were adjusted using the FDR method (Benjamini and Hochberg 1995).

## Results

### Strong interpopulation variability in seed production, germination and persistence

The three geographically distant zones across the Catalan coast (Cap de Creus, Costa Brava and Maresme) have an annual precipitation from 558 to 621 mm and an annual mean temperature around the 16 °C (Online Resource 1). Despite the similar environmental conditions, the multivariate analysis of the populations revealed significant effects for zones and populations (*P* < 0.001) (Fig. 1b). The relative position of the different populations on the multidimensional scaled plot, reveal that some distant populations such as M3 and CA1 are more similar than close populations such as M3 and M1 in terms of the 9 seed traits considered (Fig. 1b).

**Fig. 2:**
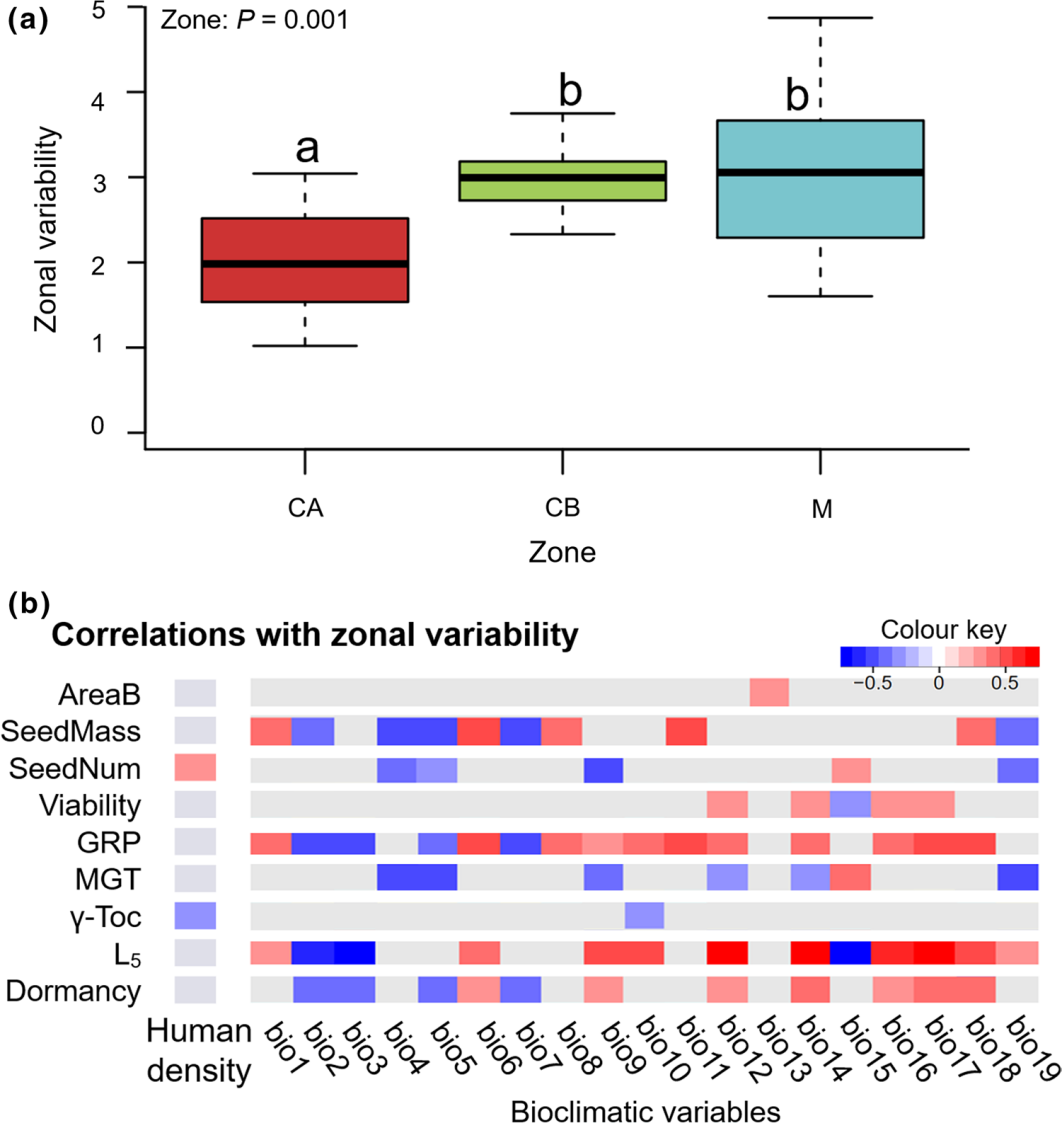
Trait values (mean ± SE, *n* = 5) for the nine relevant seed traits in all nine *C. edulis* populations from three distant zones: Cap de Creus (CC), Costa Brava (CB) and Maresme (M). Different capital letters reflect differences between zones, whereas different lowercase letters reflect significant differences between populations

Significant differences between zones and populations were found considering almost all measured seed parameters when contrasting not only distant but also near populations (Fig. 2b, Online Resource 2). Considering the three geographically distant zones, seeds from the populations at Costa Brava (CB) showed significantly higher seed mass and viability percentages compared to Maresme and Cap de Creus but high variability was found between populations within each zone (Fig. 2b, d). Significantly lower germination percentages were found in Cap de Creus seeds although big variability was found between populations, from 25 to 80% of germinated seeds (Fig. 2E). Costa Brava seeds showed significantly lower mean germination time (about 2 weeks), whereas the mean germination time for seeds from other populations raised to 30 days (Fig. 2f). The highest percentage of dormancy was found in a Costa Brava population but high variability found within populations, from 20 to 60% of dormant seeds (Fig. 2i).

Considering the different near and distant populations, post-hoc tests of seed traits revealed that even close populations have different seed area, seed mass, total seeds per fruit, seed viability, germination percentage, mean germination time, γ-tocopherol, L_5_ and dormancy percentages (Fig. 2). The smallest seeds were found in M1 with an average seed mass of 19.44 ± 0.82 μg and a basal seed area of 0.81 ± 0.04 mm^2^ whereas the heaviest seeds were found in a population located at *ca.* 40 km, CB2, with 28.04 ± 0.01 μg, and a basal area of 1.00 ± 0.02 mm^2^ (Fig. 2a, b). Besides seed morphology, near *C. edulis* populations also differed in seed production, viability, germination, biochemistry and persistence (Fig. 2, Online Resource 2). Total seeds per fruit maximum difference between populations were found between two close populations: CB3 and CB1 and ranged from 57.73 ± 42.80 to 2478.45 ± 345.32 seeds respectively. Despite the huge standard error, those differences resulted significant (Fig. 2c), showing a more than 40 times higher average seed production at less than 1 km of distance. Seed viability ranged from 71 to 88% but germination ranged from 22 to 80% across populations, arising differences in the percentage of dormant seeds between populations (Fig. 2i). The most abundant tocochromanol found, γ-tocopherol, also differed significantly between near populations (Fig. 2g). Seeds γ-tocopherol concentration ranged from 5.43 ± 0.44 in CC2 to 7.55 ± 0.30 mg gDW^−1^ in CB1 (Fig. 2g), whereas no significant differences were found in its successor, α-tocopherol, that remained around 0.27 ± 0.01 mg gDW^−1^ (Online Resource 2). Longevity analysis revealed differences between zones with M2 showing strongly decreased seed resistance to deterioration in terms of L_5_ (Fig. 2h).

**Fig. 3:**
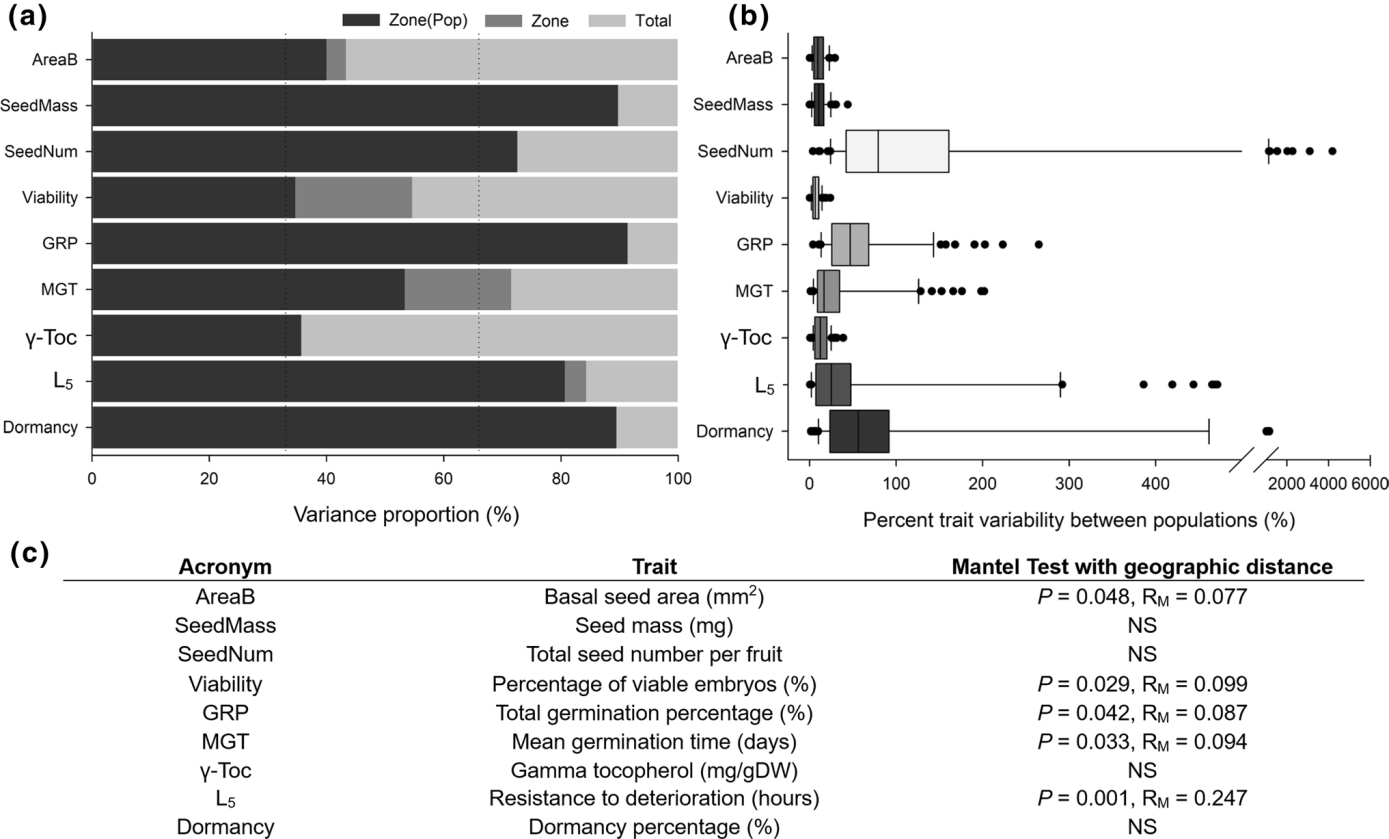
**a** Relative variance decomposition at the population and zone-levels for the different traits. The 33 and 66% thresholds are given by the dashed lines for the nine selected seed traits of *C. edulis*. **b** Boxplot of percent trait variation for the nine selected seed traits of *C. edulis* between diferent populations. **c** Acronyms, complete trait names, and Mantel Test results for the correlation between geographic variation with trait variation. *NS* non-significant, *R*_*M*_ mantel statistic

The variance structure revealed differences in the proportion of variance explained by the different factors across traits (Fig. 3a). Differences among populations captured on average, 35–90% of the total variability, while the zone factor added an additional 0–20% to the total proportion of variability explained. However, only two traits—seed viability and mean germination time—showed a proportion of variability explained by the zone factor superior to 15% (Fig. 3a).

When traits variability was translated into percent trait variability between populations, different patterns considering the different traits type were observed (Fig. 3b). The strongest percent trait variability between populations was found in total seed number per trait (up to 6000% mean values of change) whereas the smallest percent trait variability was found in seed morphology traits (up to 20% mean values). Seed germination and persistence showed also a disparate variation from 0 to 400%. Finally, γ-tocopherol content and seed viability showed a small percent variation (under 20%) (Fig. 3b).

### Geographic distance, bioclimatic variables and human density influence trait variability

Correlation of geographic with trait’s distances across populations did not result in any strong correlation in most of the studied traits, as revealed by the Mantel tests (Fig. 3c). Poor or non-significant correlation coefficients for the Mantel test were also obtained when contrasting geographic distance with the multivariate scaled seed traits distance between populations (Online Resource 2).

**Fig. 4:**
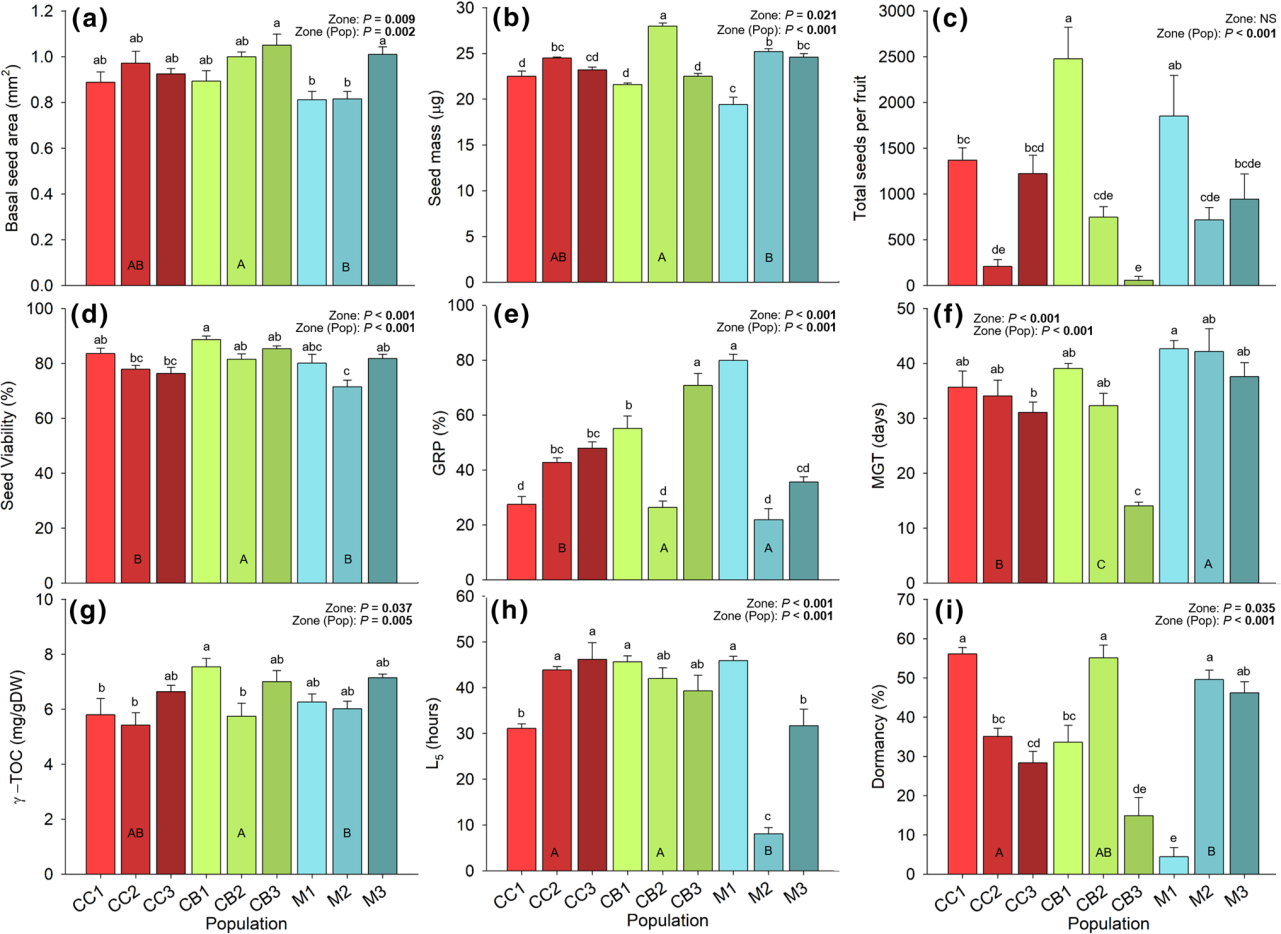
**a** Comparative of the zonal variability estimated as the distances to zonal centroid for the different populations at the zones: Cap de Creus (CA), Costa Brava (CB) and Maresme (M) using the nine selected seed traits. Different letters reflect significant differences between zones. **b** Human density and bioclimatic variables correlations with zonal variability (centroid distance) of the nine seed traits of *C. edulis* among the different populations. Coloured correlations are significant and blue to red palette represents the *R*^2^ coefficient following the colour key. Complete bioclimatic variables names can be found in http://www.worldclim.org/bioclim

Despite the high geographic variability found in almost all seed traits, this was not homogeneous among zones. The variability found between the near populations in the area of Cap de Creus, the zone with lower human density, was significantly lower than the variation between near populations in the other zones (Fig. 4a). When decomposing zonal variability among traits, seed number variability within a zone showed a positive significant correlation with human density, suggesting that populations in areas with a higher human presence may have stronger variability in this trait (Fig. 4b). The accumulation of γ-tocopherol correlated inversely to human density, suggesting that the lower human density, the higher γ-tocopherol variability. Seed traits variability was contrasted with the 19 bioclimatic variables from the WorldClim model and revealed strong and significant correlations with most traits (Fig. 4b). The bioclimatic variables with the strongest significative correlations (*P* < 0.05, *R*^2^ > 0.6 and *R*^2^ < − 0.6) with traits variability were: bio3 (isothermality), bio12 (annual precipitation), bio14 (precipitation of the driest month), bio15 (precipitation seasonality), bio16 (precipitation of the wettest quarter) and bio17 (precipitation of the driest quarter). Among those variables, some showed a clear negative correlation with most traits’ variability. Mean diurnal (bio2) and annual range (bio7), isothermality (bio3), temperature seasonality (bio4) and the maximum temperature of the warmest month (bio5) correlated inversely with zonal traits variability. In the other hand, annual mean temperature (bio1), minimum temperature of the coldest month (bio6), the temperature of the wettest (bio8) and the coldest (bio11) year quarters and precipitation of the wettest (bio16), driest (bio17) and warmest year (bio18) quarters showed strong positive correlations with zonal traits variability (Fig. 4b). Higher variability was therefore observed in zones with higher temperature and precipitation among the year, and lower temperature variation within days and seasons. The strongest correlation found was a positive correlation between the precipitation of the driest month (bio14) and resistance to deterioration (L_5_) variability (*P* < 0.05, *R*^2^ = − 0.71). This trait showed also strong significant (*P* < 0.05, *R*^2^ > 0.63) correlations with different bioclimatic variables: isothermality (bio3) and precipitation seasonality (bio15) correlated with decreased L_5_ variability, whereas annual precipitation (bio12), precipitation of the wettest (bio16) and the driest (bio17) year quarters correlated with increased L_5_ variability (Fig. 4b). Altogether, the higher L_5_ variability was found in sites with higher and less variable precipitation among the year.

## Discussion

### Variability in seed traits in geographically near populations

Intraspecific variation is considered as a key factor mediating the effects of individuals on community structure and ecosystem functioning (Des Roches et al. 2018). However, intraspecific variation may be found not only between individuals but also between different populations or even regions (Evangelista et al. 2019). In this study we obtained percentages of variation across the geographic space, revealing the existence of surprisingly high seed traits interpopulation variability in an invasive species, regardless of geographic distance.

Seed traits types showed a differential degree of trait variation, suggesting different traits sensitivity, supporting hypothesis 2 that expected high interpopulation variability in key seed traits determining soil seed bank dynamics such as seed production, germination, and persistence. Among the trait groups that showed the strongest geographic variation—seed production, seed germination and seed persistence—seed production is known to vary strongly in some species in the temporal scale (i. e., interannual variability) (Moreira et al. 2014; Bogdziewicz et al. 2019). Few studies have considered spatial variation in seed production traits, but for example, the study from Wright et al. (2005) evaluated both temporal and spatial variation and pointed out that spatial variation in seed fall was greater than temporal variation among years for all the 108 studied species from Panama for 15 years. This spatial variation in seed production could be a result of the environmental conditions, genetic diversity or also adaptation to pollinators and seed predator regimes (Linhart et al. 2014; Moreira et al. 2014; Mitchell et al. 2017). Besides high seed production geographic variability, germination, and persistence traits showed also a high spatial variability. Mother plant water stress has been described to compromise seed viability and dormancy in *Astragalus nitidiflorus* growing in the Mediterranean area (Segura et al. 2015). As fruit ripening occurs during the harsh Mediterranean summers in *C. edulis*, mother plant water stress could be the cause of the observed variability in seed traits among populations. However, only mean germination time showed an inverse significant correlation with annual precipitation and precipitation of the driest month, suggesting that summer low water availability may impact variability of mean germination time for this species. Intrapopulation variability in germination is common as a result of genetic factors and climate variability during seed ripening (Baskin and Baskin 1973). Strong regional variability in seed germination was found in *Nothofagus glauca* when contrasting 9 populations from two differentiated regions in Chile revealing higher variability within regions than between them (Santelices Moya et al. 2017). Germination variability together with high intraspecific variability in persistence traits may maximize plant long-term fitness through a bed-hedging strategy, ensuring regeneration at different time scales (Tielbörger et al. 2011; Huang et al. 2016).

Geographic variability in the different analysed traits related to *C. edulis* sexual reproduction may have implications on the dynamics of the invasive populations. *C. edulis* populations located at less than 1 km, showed differential seed production, seed mass, seed germination percentage, mean germination time, seed longevity, and dormancy percentages, and these differences between populations may suppose differential soil seed bank dynamics at the different sites. The population of Carrer del Golf (CB2) for example, showed a similar seed trait’s profile to Far de s’Arenella (CC1) and Can Teixidor (M2), located at 40 and almost 100 km of distance respectively. Those three populations have high proportions of dormant viable seeds (Figs. 2, 3). Besides, the *ca.* 1000 seeds produced per fruit in those populations contribute to forecast the formation of a soil seed bank at the described locations. Contrarily, the population Puig Sa Guilla (CB3) that showed a small seed production (less than 100 seeds per fruit), have the highest germination percentages and the lowest germination time, revealing low dormancy ratios, similarly to the population at Ca l’Antic (M1). Variability in achene traits was assessed in *Fallopia x bohemica* in different French populations, revealing differential dispersion strategies between populations, which may facilitate the colonization of contrasting environments (Lamberti-Raverot et al. 2019). Similarly, the seed traits interpopulation variation in *C. edulis* determines different strategies regarding seed bank dynamics. Some of the analysed *C. edulis* populations showed a persistence strategy with the formation of a soil seed bank whereas others showed a more expansive strategy (high germination and low dormancy rates). Interpopulation trait variation, including traits related to sexual reproduction, should be included in ecological models to predict species distribution (Albert et al. 2011; Banitz 2019; Snell et al. 2019) but also to predict invasive species potential distribution and impact.

### Contributors to seed traits variability: geographic distance, bioclimatic conditions and human density

When unravelling the determinants of intraspecific seed trait variability in *C. edulis*, population, bioclimatic conditions and human density seem to play a role. The *C. edulis* populations separation determines differences in traits related to the sexual reproduction independently of the geographic distance between them, as revealed the absence of strong correlations at the Mantel test, the high percentage of variance explained by the population factor and the differences in mean trait values between close populations, revealing that there are no identical populations in terms of seed traits as the nearest populations can be the less similar. We, therefore, reject the hypothesis 1 that expected low differences between close populations, in contrast with higher interpopulation variability between distant populations. This strong interpopulation variability regardless of geographic distance may hinder species modelling and may have different causes.

As hypothesized (hypothesis 3), human density may be one of the causes of increased interpopulation seed production variability in *C. edulis*. A recent study with the clonal invader *Plantago lanceolata* revealed that in the invasive range, repeated, long-distance, human-mediated introductions promoted high genetic diversity (Smith et al. 2020). As in *P. lanceolata*, the human presence in the invasive range may also play a role in *C. edulis* variability, as the variability in *C. edulis* seed traits found between the different close populations was different between the three contrasted zones with a human density gradient. The area with the lowest human presence, the zone of Cap de Creus, was the zone where the smallest variation in *C. edulis* seed traits was registered. The increased number and variability of novel habitats generated by urbanization (Irwin and Bockstael 2007) and disturbance (Kumordzi et al. 2019), and also the increased chance of repeated human-mediated introductions (Smith et al. 2020) may contribute to the increased seed traits variability found in *C. edulis* zones with intense anthropogenic pressure. In this way, the meta-analysis conducted by Williams et al. (2015) already exposed that the observed variability in urban plant traits, including seed traits, is linked to the consistency and strength of urban stressors. This result, in concordance with the framework presented by Gaertner et al. (2017), suggests that anthropogenic pressure in urban areas may exacerbate the invasive species impacts not only by acting as launching sites but also by increasing its intraspecific variability of species within urban environments. In the case of *C. edulis*, the study of Lechuga-Lago et al. (2017) revealed that there is a strong link between invasion and urban areas, as urbanisation assist *C. edulis* invasion, which in turn increase the impact of urbanisation. This study suggests that *C. edulis* invasion of urban soils is facilitated due to soil degradation, which allows the establishment of this species and hinders some native species establishment. Besides, higher human density may promote increased propagule pressure in species with horticultural interests, which increases the probability of a successful establishment (Willi and Van 2019). The particular bioclimatic conditions that the species encounter in each population also contributes to seed traits spatial variability, as most of the analysed seed traits correlated with bioclimatic variables. Environmental variables influenced for instance *Fagus sylvatica* seed production variability, as the mean temperature was found to be correlated with seed production (Bogdziewicz et al. 2019). Moreover, the high seed traits variability among invasive Chilean *Taraxacum officinale* populations appeared to be highly correlated with variation in rainfall (Molina-Montenegro et al. 2018) revealing the importance of environmental conditions and seasonality in determining seed traits variability. Recently, the study of global gradients for intraspecific trait variation with almost 3000 species revealed pronounced associations with climate (Kuppler et al. 2020). Despite the idiosyncrasy of species-specific associations across gradients, in general, lower variation was observed in colder areas and higher in drier areas. In concordance with this study, seed traits variation in *C. edulis* was generally higher in zones with higher temperatures. However, increased interpopulation variability was observed with higher precipitation along the year. This contradicts the hypothesis 3 that intrapopulation variability may be higher with lower temperatures in winter and low water availability in summer, which constitute two known stressful periods for species inhabiting the Mediterranean climates, including *C. edulis* (Fenollosa et al. 2017). Stressful conditions may not induce seed trait variation in *C. edulis*. In this way, our study supports the stress-reduced variability hypothesis that states that variability decreases with abiotic conditions that may generate stress (Klopfer and MacArthur 1961). Changes in the global bioclimatic conditions may have an impact on seed traits variability of *C. edulis*. However, reduced pluviometry expected in a global change framework (Christensen et al. 2007) may exhort contrary effects on seed traits variability in contrast to increased temperatures. Further research must be addressed to assess global change effects on *C. edulis* seed traits variability.

### Geographic intraspecific variation in invasive species: management implications

Knowledge regarding the intrapopulation variation across the geographic space may enhance our ability to predict ecological impacts of invasive species across different invaded areas. The found variability in seed traits between *C. edulis* populations may have important implications for this invasive species management. The work developed by Loddo et al. (2019) that evaluated variability in the invasive species *Abutilon theophrasti* seed traits demonstrated that achieving a better knowledge of interpopulation variability can allow specific control strategies to be designed, facilitating the development of new management tools. Likewise, germination rates of the Alexandra palm were found to potentially better inform of management strategies for the control of this species (Wen 2019). Considering the obtained results in *C. edulis*, the differential seed behaviour may determine differential requirements in soil management after eradication. Populations such as CB2, CC1 and M2, with high seed dormancy rates may require management cost in the long term after eradication as recruitment may be expected from the soil seed bank. The reason why those populations, with similar bioclimatic conditions and human density to the others within their zone, behave this way is uncertain and may require further research. However, the ability to form persistent seed banks might contribute substantially to determine the invasion potential of alien plants in their new distribution ranges, given the role of seed banks as sources of propagules, genetic diversity, and in spreading the risk of germination failure over time (Gioria et al. 2019). Therefore, the inclusion of soil seed bank dynamics analysis during prioritization of eradication zones may help to develop cost-effective management strategies, by increasing investment in post-eradication management in populations with high seed longevity and prioritizing populations with strong seed production, as those populations may have an increased invasive potential (Pyšek and Richardson 2007).

Our study quantified the interpopulation variability in traits related to the sexual reproduction *C. edulis* between near (< 4 km) and distant populations (> 40 km) and revealed high geographic variability in seed production, germination and persistence traits. The observed variability in the *C. edulis* seed production, germination and dormancy rates in some populations suggested differential soil seed bank dynamics that may require differential strategies for cost-effective management. Seed traits interpopulation variability was found to be influenced by bioclimatic conditions suggesting a potential impact of climate shifts. Moreover, increased human density correlated with higher interpopulation variability in seed production suggesting a role of high anthropogenic pressure in seed traits variability which may contribute to exacerbate the invasive species impacts in urban areas. Interpopulation trait variation should be included in ecological models and will be important for assessing invasive species responses to environmental heterogeneity and global change.

## Supplementary Information

Below is the link to the electronic supplementary material.

Supplementary file1 (XLSX 12 KB) Online Resource 1 Bioclimatic variables for all 9 analysed populations. https://www.worldclim.org/data/bioclim.html.

Supplementary file2 (DOCX 21 KB) Online Resource 2. List of measured traits with their acronyms, ecological relevance, potential for invasiveness and Mantel Correlation coefficient (RM) and P-values for the Mantel test contrasting the distance between populations of each trait or trait group versus the geographic and bioclimatic distances. NS = Not significant.

Supplementary file3 (XLSX 23 KB) Online Resource 3. Mean values per population and P-values of the Zone and Zone (Population) effects in the ANOVA for all measured traits. NS = Not significant. Data are shown as Mean ± SE, N = 5.
