# Supplementary material for: Geographic patterns of seed trait variation in an invasive species: how much can close populations differ?
Source: Oecologia. 2021 Jul 3;196(3):747–61. doi: 10.1007/s00442-021-04971-2 (PMC8292299; doi:10.1007/s00442-021-04971-2)
Supplement: Supplementary file 2 — Supplementary file2 (DOCX 21 KB) Online Resource 2. List of measured traits with their acronyms, ecological relevance, potential for invasiveness and Mantel Correlation coefficient (RM) and P-values for the Mantel test contrasting the distance between populations of each trait or trait group versus the geographic and bioclimatic distances. NS = Not significant. [file 442_2021_4971_MOESM2_ESM.docx]

# Online Resource 2

List of measured traits with their acronyms, ecological relevance, potential for invasiveness and Mantel Correlation coefficient (R_M_) and *P*-values for the Mantel test contrasting the distance between populations of each trait or trait group versus the geographic distances. NS = Not significant

|  |  |  |  |  |  |  |
| --- | --- | --- | --- | --- | --- | --- |
| **Trait Type** | **Trait** | **Acronym** | **Ecological relevance** | **Potential for invasiveness** | **Geographic distance** | |
| Morphometry | Basal seed area (mm^2^) | AreaB | Descriptors of seed size and shape, directly related to dispersal as they influence abiotic and biotic travel distance, determining how and how efficiently seeds disperse. | Seed morphological traits may determine differential dispersion strategies that can favor adaption and colonization of different novel habitats. | *P* = 0.048, RM = 0.077 | P = 0.018, RM = 0.108 |
| Morphometry | Basal seed perimeter (mm) | PerimB |  |  | *P* = 0.031, RM = 0.104 |  |
| Morphometry | Major basal diagonal (mm) | MajorB |  |  | *P* = 0.021, RM = 0.104 |  |
| Morphometry | Minor basal diagonal (mm) | MinorB |  |  | NS |  |
| Morphometry | Lateral seed area (mm^2^) | AreaL |  |  | NS |  |
| Morphometry | Lateral seed perimeter (mm) | PerimL |  |  | *P* = 0.046, RM = 0.071 |  |
| Morphometry | Major lateral diagonal (mm) | MajorL |  |  | *P* = 0.045, RM = 0.076 |  |
| Morphometry | Minor lateral diagonal (mm) | MinorL |  |  | NS |  |
| Morphometry | Seed mass (mg) | SeedMass |  |  | NS |  |
| Morphometry | Dry seed mass (mg) | DrySeedMass |  |  | NS |  |
| Biochemistry | Gamma tocopherol (mg/gDW) | γ-Toc | Tocopherol seed content has a critical role in maintaining seed viability and protecting lipids from oxidation during germination and early seedling growth. | Increased antioxidant content and reduced seed water content may contribute to seed survival within the soil seed bank and during germination. | NS | *P* = 0.001, RM = 0.213 |
| Biochemistry | Alpha tocopherol (mg/gDW) | α-Toc |  |  | NS |  |
| Biochemistry | Total tocopherols (mg/gDW) | Toc |  |  | NS |  |
| Biochemistry | Seed water content (%) | WC | Seed water content is related with deterioration and molecular mobility. |  | *P* = 0.003, RM = 0.145 |  |
| Biochemistry | Imbibed seed water content (%) | Imb_WC |  |  | *P* = 0.001, RM = 0.538 |  |
| Viability | Percentage of viable embryos (%) | Viability | Account for the percentage of seeds that are viable. | Higher proportion of viable seeds may contribute to increased propagule pressure and increased recruitment, but also contributes to seed survival with time before germination. | *P* = 0.029, RM = 0.099 | *P* = 0.0.001, RM = 0.239 |
| Viability | Percentage of patchy embryos (%) | Patchy | Account for the percentage seeds with different embryo types with decreased vigor. |  | *P* = 0.007, RM = 0.157 |  |
| Viability | Percentage of weakly embryos (%) | Weakly |  |  | *P* = 0.007, RM = 0.167 |  |
| Viability | Percentage of patchy-weakly embryos (%) | PatchyWeakly |  |  | NS |  |
| Viability | Percentage of death embryos (%) | Death |  |  | NS |  |
| Viability | Percentage of aborted seeds (%) | Aborted | Account for the percentage of produced seeds that are aborted. |  | NS |  |
| Viability | Percentage of dying embryos (%) | Dying | Account for the percentage of non-death seeds with different embryo types with decreased vigor. |  | *P* = 0.001, RM = 0.232 |  |
| Production | Fruit weight (g) | FruitWeight | Define the seed production in terms of weight or number determining propagule pressure. | Higher seed production increases the propagule pressure and potential recruitment. | NS | NS |
| Production | Total seed weight per fruit (mg) | SeedsWeight |  |  | NS |  |
| Production | Total seed number per fruit | SeedNum |  |  | NS |  |
| Persistence | Resistance to deterioration (hours) | L_5_ | Define the seeds potential resistance to ageing. | Long-lived and dormant seeds may allow determine the persistence potential of the soil seed bank. | *P* = 0.001, RM = 0.247 | *P* = 0.001, RM = 0.245 |
| Persistence | Medium longevity (hours) | L_50_ |  |  | *P* = 0.003, RM = 0.171 |  |
| Persistence | Lethal ageing time (hours) | L_95_ |  |  | NS |  |
| Persistence | Dormancy percentage (%) | Dormancy | Determine the percentage of the dormant state that may contribute to seed persistence. |  | NS |  |
| Germination | Total germination percentage (%) | GRP | Determine the percentage of seeds that may germinate when encountering the optimal conditions. | High germination rates and a fast germination increase the potential recruitment. | *P* = 0.042, RM = 0.087 | *P* = 0.008, RM = 0.132 |
| Germination | Mean germination time (days) | MGT | Define the germination timing. Germination timing optimizes the fitness of seedlings by delivering germinated seeds at the best moment. |  | *P* = 0.033, RM = 0.094 |  |
| Germination | Mean germination rate(seeds/day) | MGR |  |  | NS |  |
| Germination | Germination speed percentage (%) | GSP |  |  | NS |  |
| Germination | Time required for 50% germination (days) | T_50_ |  |  | *P* = 0.002, RM = 0.162 |  |
| Germination | Uncertainty index (bits) | UNC | Define seed germination synchronization and variability. Variability is associated with seedling survival chance in heterogeneous framework. |  | *P* = 0.043, RM = 0.094 |  |
| Germination | Synchronization index (SYN) | SYN |  |  | NS |  |
| Germination | Germination variance | VGT |  |  | *P* = 0.003, RM = 0.188 |  |
| Germination | Germination standard deviation | SDG |  |  | *P* = 0.019, RM = 0.112 |  |
| Germination | Coefficient of variation (%) | CVG |  |  | NS |  |
